# Laminar Distribution of Neurochemically-Identified Interneurons and Cellular Co-expression of Molecular Markers in Epileptic Human Cortex

**DOI:** 10.1007/s12264-018-0275-x

**Published:** 2018-08-31

**Authors:** Qiyu Zhu, Wei Ke, Quansheng He, Xiongfei Wang, Rui Zheng, Tianfu Li, Guoming Luan, Yue-Sheng Long, Wei-Ping Liao, Yousheng Shu

**Affiliations:** 1 State Key Laboratory of Cognitive Neuroscience and Learning and IDG/McGovern Institute for Brain Research, Beijing Normal University, Beijing, 100875 China; 2 College of Pharmaceutical Sciences, Brain Institute, Capital Medical University, Beijing, 100069 China; 3 Department of Neurosurgery, Epilepsy Center, Sanbo Brain Hospital of Capital Medical University, Beijing Key Laboratory of Epilepsy, Epilepsy Institution, Beijing Institute for Brain Disorders, Beijing, 100093 China; 4 Institute of Neuroscience and Department of Neurology of the Second Affiliated Hospital of Guangzhou Medical University, Key Laboratory of Neurogenetics and Channelopathies of Guangdong Province and the Ministry of Education of China, Guangzhou, 501260 China

**Keywords:** Interneuron, Epilepsy, Human cortex, Cell type, Immunostaining, Parvalbumin, Somatostatin, Tyrosine hydroxylase, Neuropeptide Y, Cholecystokinin

## Abstract

Inhibitory GABAergic interneurons are fundamental elements of cortical circuits and play critical roles in shaping network activity. Dysfunction of interneurons can lead to various brain disorders, including epilepsy, schizophrenia, and anxiety. Based on the electrophysiological properties, cell morphology, and molecular identity, interneurons could be classified into various subgroups. In this study, we investigated the density and laminar distribution of different interneuron types and the co-expression of molecular markers in epileptic human cortex. We found that parvalbumin (PV) and somatostatin (SST) neurons were distributed in all cortical layers except layer I, while tyrosine hydroxylase (TH) and neuropeptide Y (NPY) were abundant in the deep layers and white matter. Cholecystokinin (CCK) neurons showed a high density in layers IV and VI. Neurons with these markers constituted ~7.2% (PV), 2.6% (SST), 0.5% (TH), 0.5% (NPY), and 4.4% (CCK) of the gray-matter neuron population. Double- and triple-labeling revealed that NPY neurons were also SST-immunoreactive (97.7%), and TH neurons were more likely to express SST (34.2%) than PV (14.6%). A subpopulation of CCK neurons (28.0%) also expressed PV, but none contained SST. Together, these results revealed the density and distribution patterns of different interneuron populations and the overlap between molecular markers in epileptic human cortex.

**Electronic supplementary material:**

The online version of this article (10.1007/s12264-018-0275-x) contains supplementary material, which is available to authorized users.

## Introduction

In the cerebral cortex, non-pyramidal GABAergic interneurons are involved in cortical information-processing and high-order cognitive functions. Though non-pyramidal cells (20%–30%) are much less numerous than the main output neurons, pyramidal cells (70%–80%) [1–3], in the whole population of cortical neurons, they are more extensive and complex. Interneurons can be classified depending on their morphology, intrinsic membrane properties, and synaptic connectivity and dynamics. Distinct interneuron subtypes can be also identified by the expression of specific molecular markers, such as parvalbumin (PV), somatostatin (SST), tyrosine hydroxylase (TH), vasoactive intestinal polypeptide, ionotropic serotonin 5-hydroxytryptamine 3a receptor, nitric oxide synthase, cholecystokinin (CCK), and neuropeptide Y (NPY) [4–6]. Although some of them (e.g. CCK) are also expressed by a subpopulation of glutamatergic pyramidal cells [7], neurons labeled with these markers are most likely to be GABAergic cells in the neocortex [5, 8, 9] and possess distinct electrophysiological and morphological features. For example, PV-expressing neurons show a fast-spiking firing pattern and send axons to innervate the perisomatic regions of pyramidal cells, while SST-containing neurons show a low-threshold spiking firing pattern and innervate the distal apical dendrites of pyramidal cells [10–13]. Because of the fundamental role of GABAergic interneuron in providing inhibitory control of cortical network, changes in interneuron circuitry and alterations of GABAergic transmission in the cortex can lead to disorders of cognition and emotion, such as schizophrenia, anxiety, and epilepsy [14–17].

An epileptic seizure is a paroxysmal alteration of function caused by excessive, hyper-synchronous discharge of neurons and abnormal network activity in the brain. Although numerous pathogenic conditions can result in epilepsy along with brain dysfunction [14, 18, 19], its pathophysiology is generally considered to be a distortion of the normal, well-balanced excitation (E) and inhibition (I) in the brain [20]. A genetic or acquired E–I imbalance can result from changes at many levels, from genes and subcellular signaling cascades to neural circuits. GABAergic interneurons are critical circuit elements in the cortex, providing inhibition in cortical networks, and thus contribute significantly to the E–I balance. Alterations in their distribution and density in the cortex, as well as changes in the co-localization of different molecular markers in interneuron subtypes may reflect the mechanisms underlying brain diseases. Previous studies have revealed an association between hippocampal GABAergic interneurons and the generation of epilepsy [21]. Changes in GABA production or GABA receptor expression have been found in epileptic tissues [22, 23]. However, the distribution and co-localization patterns of different molecular markers for GABAergic interneurons in the human epileptic cortex need to be further explored.

Among cortical interneurons, PV- and SST-expressing cells are the most abundant cell types [5, 24]. In the human cortex, PV neurons including chandelier cells and large basket cells [25] comprise ~ 20% of all GABAergic neurons [26]; SST neurons are distributed unevenly across the human cortex [25, 27, 28]. PV and SST neurons play important roles in the generation of cortical network activity, such as gamma and beta oscillations [29–31], as well as seizure-like activity [32]. NPY is a neuropeptide produced by certain types of neurons throughout the brain and by secretory cells of other systems [33, 34]. In the neocortex, NPY is expressed in a subpopulation of GABAergic neurons and is involved in brain disorders including seizure activities [33, 35]. The NPY neuron density is high in layers II, III, and VI, and in the white matter of human cortex [36]. TH is a molecular marker of midbrain dopaminergic neurons and is the rate-limiting enzyme of dopamine synthesis. Some cortical cells also express this enzyme and may reflect a unique cell type in the neocortex [37]. TH neurons in the human cortex are mainly located in deep layers and are fusiform, bipolar, or multipolar [38, 39]. Early immunostaining experiments revealed the expression of CCK in a subpopulation of GABAergic neurons in the neocortex [40]. Some of the CCK-expressing neurons in the hippocampus are basket cells targeting the perisomatic regions of pyramidal cells [41, 42]. Selective loss of hippocampal CCK-containing boutons and thus a decrease in GABA release may cause epilepsy in an animal model of temporal lobe epilepsy [40, 43]. CCK neurons in the human cortex are also positive for calretinin or reelin [7]. Since a particular cell type may express a combination of markers, it is of interest to determine whether PV and SST cells also express NPY, TH, and CCK, and determine whether cells positive for one of these markers are a subpopulation of PV or SST cells.

In this immunohistochemical study, we performed double- and triple-labeling to analyze the distribution patterns of the above molecular markers of interneurons and their co-localization in cortex from patients.

## Materials and Methods

We used epileptic tissues removed from 9 patients during brain surgery. All were associated with secondary epilepsy caused by various pathological conditions. The cortical areas removed were mainly from the frontal or temporal lobe (Table 1). Non-epileptic peri-tumor tissues were obtained from 3 patients (frontal, temporal, or parietal lobe). The Ethics Committee of Beijing Sanbo Hospital, Capital Medical University, China approved all studies. We have complied with all relevant ethical regulations relating to the use of resected human brain tissue in research. The clinical investigations were conducted according to the Declaration of Helsinki. Informed consent was given by all participants or their parents or legal guardians.

**Table 1:** Patient information

No.	Age, sex	Duration of epilepsy	Etiology	Seizure frequency	Previous treatment	Drug treatment	Surgical results	Surgical resection area
1	16 m, F	7 m	FCD 2a	Several times/d	VNS	Oxcarbazepine, Sodium valproate, Clonazepam	Seizure-free	Right temporal lobe
2	19 m, M	12 m	Sturge-Weber syndrome	2–4/m	None	Oxcarbazepine, Topiramate, Clonazepam	Seizure-free	Left anterior temporal lobe
3	14 y, M	14 y	Trauma	3/m	None	Sodium valproate	Seizure decrease	Left frontal lobe
4	16 y, F	6 y	FCD 1b	10/m	IEI	Carbamazepine, Sodium valproate	Seizure-free	Left anterior temporal lobe
5	39 y, M	30 y	FCD 2b	4-5/w	None	Sodium valproate	No record	Right frontal lobe
6	28 y, M	9 y	FCD 1b	2/m	IEI	Oxcarbazepine, Sodium valproate	No record	Left anterior temporal lobe
7	28 y, M	20 y	FCD 1b	Several times/d	None	Carbamazepine, Sodium valproate	Seizure-free	Left temporal lobe
8	8 y, M	8 y	FCD 1b	10/w	IEI	Oxcarbazepine, Clonazepam	Seizure decrease	Left anterior temporal lobe
9	8 y, M	0.5 y	FCD 1b	3–4/w	None	None	No record	Left anterior temporal lobe
10	62 y, F	–	Meningioma	–	–	–	–	Right frontal lobe
11	47 y, M	–	Melanoma	–	–	–	–	Left parietal lobe
12	62 y, F	–	Breast cancer	–	–	–	–	Left temporal lobe

m, month; y, year; d, day; w, week; F, female; M, male; focal cortical dysplasia; FCD 1b, defective horizontal lamination with lamina-specific neuronal paucity [44]; FCD 2b, dysplastic megalocytic neurons, balloon cells, delaminated cortical architecture and abnormal glial cells, mixed with neurons and glia that appear histologically normal [44]; IEI, intracranial electrode implantation; Sturge-Weber syndrome, neurocutaneous disorder with angiomas that involve the leptomeninges; VNS, vagus nerve stimulation.

Cortical tissues were removed during the course of neurosurgery for the treatment of patients with intractable epilepsy. Before the surgery, epileptogenic regions were identified by video-EEG recording and the removed regions were associated with significant abnormal spiking. Since we sought to explore the laminar distribution patterns of interneuron subtypes, we chose parts of the removed tissue blocks showing complete cortical layers (from layer I to white matter). All tissues were immersed in 3% paraformaldehyde (PFA) and 3% sucrose in 0.1 mol/L phosphate buffer (PB, 80 mmol/L Na_2_HPO_4_, 16 mmol/L NaH_2_PO_4_, pH 7.4) at 4 °C for 2 h. They were then stored in 30% sucrose in PB overnight at 4 °C for cryoprotection. Tissues were cut into 30-μm-thick sections on a freezing microtome at − 20 °C. Sucrose and residual PFA were washed out in 0.01 mol/L PBS buffer (in mmol/L: 8 Na_2_HPO_4_, 1.6 NaH_2_PO_4_, and 145 NaCl), the sections were then pre-incubated in 0.5% Triton X-100 in PB for 30 min, and blocked in 5% bovine serum albumin and 0.5% Triton X-100 in PB for 1 h at room temperature (22 °C). Sections were then incubated overnight at 4 °C and two more hours at room temperature in 0.1% Triton X-100 containing primary antibodies (Supplementary Table S1). After washing with 0.01 mol/L PBS, sections were incubated for 2 h at room temperature in 0.1% Triton X-100 containing secondary antibodies.

We chose to image non-successive sections (~ 2 mm × 2 mm) for each experiment so that no cell on the surface would be over-counted. We collected images from 3 regions of interest in separate cortical sections from each patient. The regions of interest for cell counting contained complete cortical layers (from pia to white matter) and at least 1000 NeuN-stained cells. For the co-localization of cell markers (double- and triple-staining), we selected regions of similar size but without NeuN staining. Images were captured on a laser scanning confocal microscope (A1+, Nikon, Japan) with 10× and 20× objectives. The acquisition parameters were carefully adjusted to linearly display the fluorescence signals and ensure that they fell in the maximum dynamic range of the detectors. To generate Z-stacks for each marker and each cortical field, we acquired 3 images (3.5-μm intervals) at a magnification of 10× and 6 images (1.5-μm intervals) at 20×. Original images were processed with ImageJ (National Institutes of Health, USA) to adjust the brightness and contrast, and then exported as TIFF images. We used MatLab (2014a, Mathworks, USA) and ImageJ to calculate the total number of cells in regions of interest. To count the number of cells labeled by a particular marker, we set a threshold of fluorescence intensity and only those above the threshold were counted. In our experiments, it was clear that the labeled cells showed a much higher fluorescence intensity than the background. Positive cells were also determined by soma size. For instance, NeuN-labeled cells with a soma size between 5 and 35 μm in diameter were counted.

We identified cortical layers using the following characteristics of different layers, assisted by a MatLab code that determined the soma size and density of NeuN-labeled cells. Positively-labeled cells in layer I were sparse. Compared with layer III, layer II had a higher cell density but the cells were smaller. Layer IV had a higher cell density than its neighboring layers (III and V). Layer V had the largest pyramidal cells. Layer VI cells were smaller than layer V cells. The border between layer VI and the white matter was determined by a sharp decrease in cell density.

## Results

### Specificity of Antibodies

**Fig. 1:**
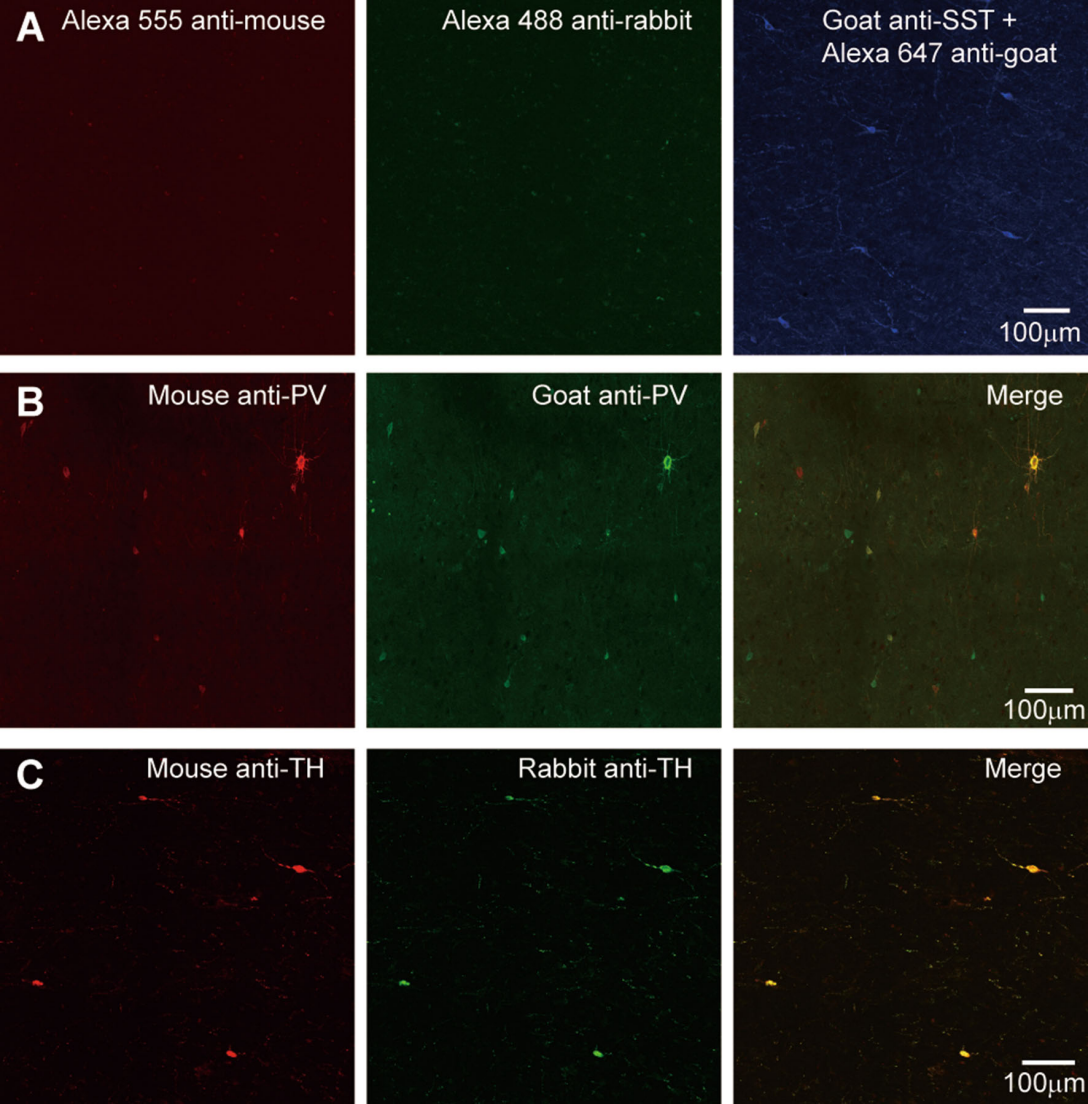
Control experiments assessing antibody specificity. **A** Goat anti-SST was used as the only primary antibody and followed by the three secondary antibodies. Only one channel showed positive signals (Alexa 647 anti-goat). **B** Double-labeling of mouse and goat anti-PV. Note that most of the labeled cells were positive for both primary antibodies. **C** Double-labeling of mouse and rabbit anti-TH. All labeled cells were positive for both primary antibodies. Cortical tissues were from patient #9 (8 years old).

We performed control experiments to confirm the specificity of the primary and secondary antibodies (Supplementary Table S1). We followed the protocols described in the Methods but with some changes in the procedures. To assess the specificity of the secondary antibodies, for each primary antibody (NeuN, PV, SST, CCK, NPY, and TH), we applied all three secondary antibodies. We found that only the corresponding secondary antibody showed positive signals (Fig. 1A, SST primary antibody). Next, we replaced the primary antibody with bovine serum albumin and applied all the three secondary antibodies and observed no staining. In addition, we performed double-labeling using two distinct primary antibodies for PV (goat and mouse anti-PV) or TH (rabbit and mouse anti-TH) and assessed the overlap of labeled cells. The percentages of cells positive for both antibodies were 78% in goat and 90% in mouse anti-PV-labeled cells (Fig. 1B). For TH staining, the two primary antibodies labeled the same population of cells (completely overlapped, Fig. 1C).

### Laminar Distribution and Density of PV and SST Neurons

**Fig. 2:**
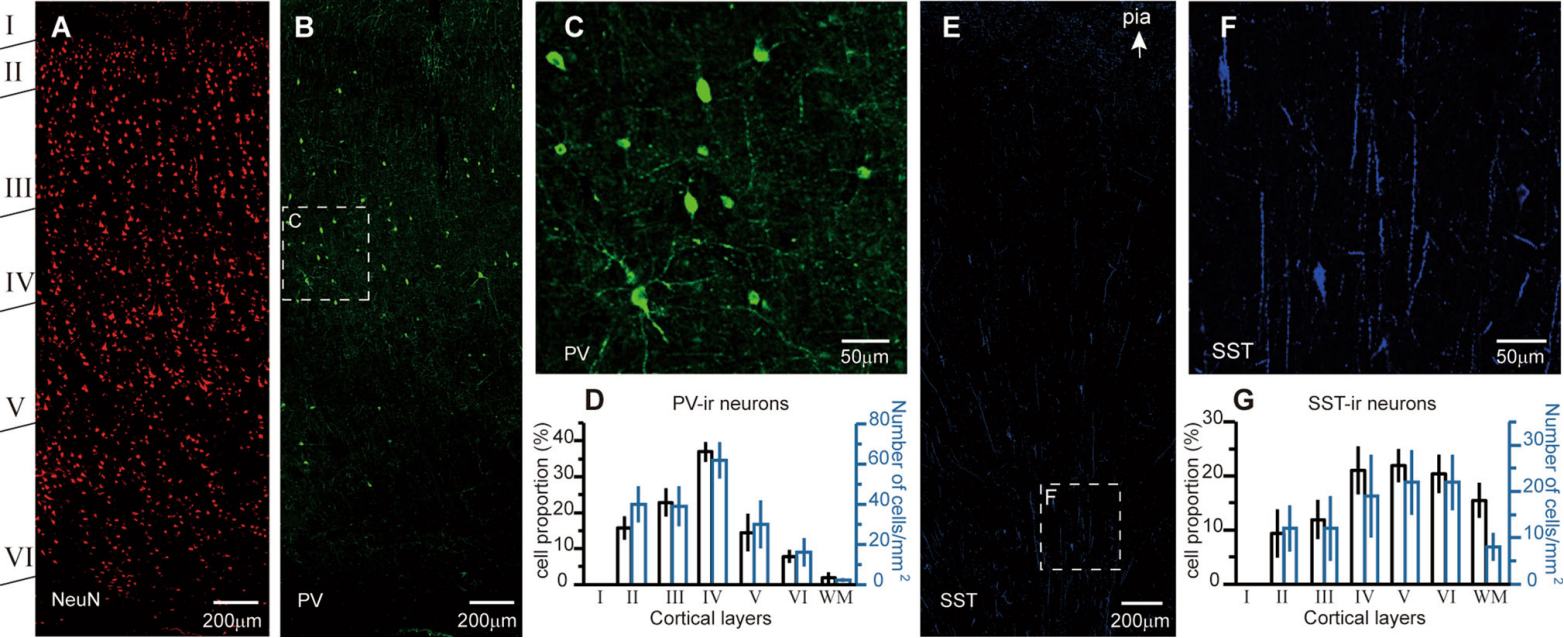
Laminar distribution of PV-ir and SST-ir neurons. **A** NeuN immunostaining showing the identification of cortical layers I to VI and the white matter. Tissue was from the left frontal lobe of patient #3 (14 years old). **B** PV labeling in the same section as in **A**. **C** Higher magnification of the boxed area in **B**. **D** Group data showing the percentage of PV-ir cells among the total PV-positive population (black, patients 1, 3, 4, 7, 8, and 9) and their density in each layer (blue, patients 7–9). **E** Distribution of SST-ir neurons in the right temporal lobe of patient #1 (1.3 years old). **F** Higher magnification of the boxed area in **E**. **G** As in **D**, but for SST-ir cells. Error bars denote SEM.

In immunohistochemical experiments, we used NeuN as the neuronal marker since it is expressed by most neurons throughout the central nervous system and has been used as a neuronal marker in previous studies [45–47]. Based on double-staining for NeuN and different neurochemical markers, we determined the total number of neurons in the regions of interest as well as the distribution and density of interneuron subpopulations (Fig. 2A). In these experiments, we used tissues from patients #1, 3, 4, 7, 8 and 9 (Table 1). We performed staining using antibodies to all the molecular markers in cortical sections from each patient. We also analyzed the cell density (number of cells per mm^2^) in different layers and the white matter.

Labeling with antibodies revealed the distribution patterns of neurons immunoreactive (ir) for PV or SST across cortical layers (Fig. 2 and Tables 2, 3, 4 and 5). We found that PV-ir neurons comprised 7.16% ± 0.54% (mean ± SEM) of NeuN-labeled gray-matter neurons (*n *= 26,759 cells from 6 patients) and located in all cortical layers except layer I, with layer IV displaying the highest density (Fig. 2A–D and Tables 2, 3). And the density of PV-ir neurons in gray matter was 34 ± 7 per mm^2^ (*n *= 1632 PV cells). Moreover, PV-ir neurons were rarely found in the white matter. The PV-ir neurons were most likely basket cells or chandelier cells, as suggested in previous studies by their somatic morphology and axonal arborization [48]. PV-ir fibers were densely distributed across all cortical layers including layer I. We also performed similar labeling in non-epileptic tissues (from patients #10–12) and found the proportion of PV-ir cells among all cortical neurons (6.99% ± 0.25%, *n *= 10,258) and their density in gray matter (34 ± 5 per mm^2^, *n* = 766) were similar to the values obtained from epileptic tissues, indicating no substantial change of PV-ir cells under epileptic conditions.

**Table 2:** Percentages of positive cells in each layer

	I	II	III	IV	V	VI	wm
*PV ep*							
1	0	14.08%	23.94%	34.32%	13.38%	7.75%	3.52%
3	0	11.40%	26.47%	34.93%	19.49%	7.72%	0
4	0	16.67%	28.04%	41.27%	9.26%	3.97%	0.79%
7	0	20.28%	20.56%	39.43%	9.86%	9.30%	0.56%
8	0	12.96%	16.05%	33.95%	23.46%	9.26%	4.32%
9	0	18.80%	22.07%	38.15%	11.17%	8.45%	1.36%
Mean	0	15.70%	22.86%	37.01%	14.44%	7.74%	1.76%
SEM	0	3.17%	3.93%	2.78%	5.27%	1.80%	1.60%
*PV non-ep*							
10	0	15.81%	19.76%	36.36%	19.37%	7.11%	1.58%
11	0	14.05%	27.57%	43.24%	9.73%	4.86%	0.54%
12	0	16.00%	23.00%	39.00%	12.00%	7.00%	3.00%
Mean	0	15.29%	23.44%	39.53%	13.70%	6.32%	1.71%
SEM	0	0.88%	3.20%	2.83%	4.12%	1.04%	1.01%
*SST ep*							
1	0	6.82%	12.50%	11.36%	26.17%	26.14%	17.05%
3	0	7.53%	9.68%	23.30%	21.15%	22.22%	16.13%
4	0	19.21%	19.49%	20.90%	16.10%	14.41%	9.89%
7	0	8.28%	10.19%	22.29%	22.93%	20.38%	15.92%
8	0	6.45%	8.60%	24.73%	21.51%	18.28%	20.43%
9	0	7.81%	10.94%	23.44%	23.44%	20.83%	13.54%
Mean	0	9.35%	11.90%	21.00%	21.88%	20.38%	15.49%
SEM	0	4.45%	3.60%	4.47%	3.06%	3.59%	3.23%
*TH ep*							
1	0	3.70%	7.41%	14.81%	25.93%	29.63%	18.52%
3	0	0	7.14%	7.21%	35.66%	35.71%	14.29%
4	0	0	5.26%	15.79%	21.05%	31.58%	26.32%
7	0	0	5.26%	15.79%	31.58%	26.32%	18.42%
8	0	0	8.33%	8.33%	33.33%	33.33%	16.67%
9	0	0	10.42%	22.92%	35.42%	20.83%	10.42%
Mean	0	0.62%	7.30%	14.14%	30.50%	29.57%	17.44%
SEM	0	1.39%	1.79%	5.24%	5.32%	4.88%	4.84%
*NPY ep*							
1	0	0	0	12.33%	14.33%	20.00%	53.33%
3	0	0	0	15.79%	21.05%	21.05%	42.11%
4	0	0	0	15.38%	7.69%	19.23%	57.69%
7	0	0	0	16.00%	16.00%	20.00%	48.00%
8	0	0	0	7.15%	14.28%	35.71%	42.86%
9	0	0	0	12.50%	16.67%	33.33%	45.83%
Mean	0	0	0	13.19%	15.00%	24.89%	48.30%
SEM	0	0	0	3.09%	3.97%	6.87%	5.59%
*CCK ep*							
1	0	15.07%	13.70%	34.25%	8.22%	24.66%	4.11%
3	0	6.45%	13.98%	32.26%	16.13%	27.96%	3.23%
4	0	21.43%	24.29%	30.00%	7.14%	15.71%	1.43%
7	0	11.49%	12.16%	32.43%	12.16%	30.41%	1.35%
8	0	11.35%	20.87%	30.81%	14.05%	28.65%	0
9	0	9.87%	11.16%	32.62%	12.02%	32.19%	2.15%
Mean	0	12.61%	16.03%	32.06%	11.62%	26.60%	2.05%
SEM	0	4.69%	4.83%	1.36%	3.12%	5.39%	1.34%

ep: epileptic; non-ep: non-epileptic; wm: white matter.

**Table 3:** Cell density in NeuN-labeled cells (counts per mm^2^)

	I	II	III	IV	V	VI	wm	gm
*PV-ep*								
1	0	33	25	47	19	10	2	23
3	0	31	52	66	39	15	0	34
4	0	32	30	63	20	6	1	29
7	0	50	51	70	28	25	1	44
8	0	52	40	74	51	18	4	43
9	0	39	36	54	23	22	3	33
Mean	0	40	39	62	30	16	2	34
SEM	0	9	10	9	12	7	1	7
*PV non-ep*								
10	0	33	37	88	49	32	2	37
11	0	49	45	76	20	13	1	39
12	0	33	41	66	16	9	1	27
Mean	0	38	41	77	28	18	1	34
SEM	0	8	3	9	15	10	1	5
*SST ep*								
1	0	5	5	6	13	13	4	8
3	0	7	6	14	15	21	6	13
4	0	20	26	34	25	25	9	23
7	0	14	15	24	24	25	15	19
8	0	13	7	14	18	16	7	13
9	0	10	15	23	34	29	9	18
Mean	0	12	12	19	22	22	8	16
SEM	0	5	7	9	7	6	3	5
*TH ep*								
1	0	1	1	3	6	9	3	4
3	0	0	2	2	5	8	2	3
4	0	0	1	2	5	6	2	3
7	0	0	2	4	8	8	4	4
8	0	0	1	1	3	3	1	1
9	0	0	2	3	8	7	5	3
Mean	0	1	2	3	6	7	3	3
SEM	0	1	1	1	2	2	1	1
*NPY ep*								
1	0	0	0	2	2	4	7	1
3	0	0	0	1	4	6	4	2
4	0	0	0	3	3	7	5	2
7	0	0	0	6	6	9	13	2
8	0	0	0	2	5	14	7	4
9	0	0	0	2	4	2	8	2
Mean	0	0	0	3	4	7	7	2
SEM	0	0	0	2	1	4	3	1
*CCK ep*								
1	0	15	11	33	7	23	1	16
3	0	8	14	33	14	28	2	21
4	0	18	11	36	26	26	1	14
7	0	19	16	33	21	58	1	24
8	0	36	41	52	28	57	0	34
9	0	19	19	38	18	54	2	29
Mean	0	19	19	38	19	41	1	23
SEM	0	8	10	7	7	15	1	7

ep: epileptic; non-ep: non-epileptic; gm: gray matter; wm: white matter.

**Table 4:** Percentage of positive cells among NeuN-labeled cells in gray matter of epileptic tissue

	1 (%)	3 (%)	4 (%)	7 (%)	8 (%)	9 (%)	Mean (%)	SEM (%)
PV	6.55	7.84	6.99	7.94	6.71	6.92	7.16	0.54
SST	2.16	2.65	3.12	2.44	2.43	2.81	2.60	0.31
TH	0.23	0.58	0.53	0.65	0.54	0.67	0.53	0.15
NPY	0.40	0.59	0.53	0.49	0.56	0.51	0.51	0.06
CCK	4.48	5.26	3.76	4.37	4.42	4.01	4.38	0.47

**Table 5:** Percentages of cells co-expressing PV or SST among the total TH-positive cell population in different patients

Patient number	PV^+^TH^+^/TH^+^	SST^+^TH^+^/TH^+^
1	26.1% (6/23)	13.0% (3/23)
3	9.67% (3/31)	38.7% (12/31)
4	12.5% (2/16)	43.8% (7/16)
5	8.33% (1/12)	40.0% (14/35)

SST-ir neurons constituted 2.60% ± 0.31% of NeuN-labeled gray-matter neurons (*n* = 35,761) and were distributed mainly in layers IV, V, VI and the border between gray and white matter (Fig. 2E–G and Tables 2, 3). The density of SST-ir neurons in gray matter was 16 ± 5 per mm^2^ (*n* = 793). SST-ir neurites were mainly located in deep layers; straight and long fibers crossing layers and perpendicular to the white matter were often observed (Fig. 2E and F).

### Laminar Distribution and Density of NPY, TH, and CCK Neurons

**Fig. 3:**
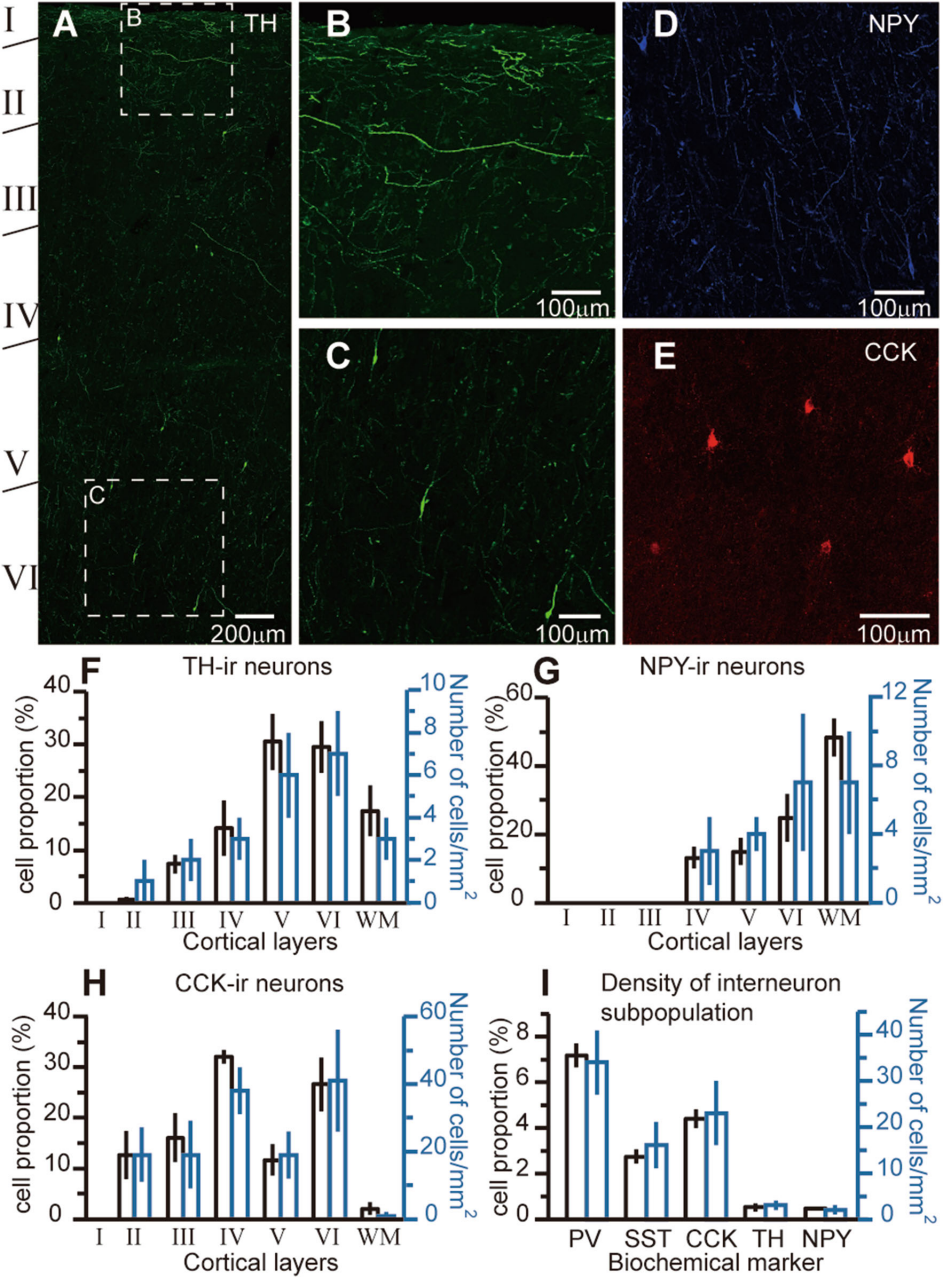
Laminar distribution of TH-ir, NPY-ir, and CCK-ir neurons. **A** Laminar distribution of TH-ir neuron across cortical layers in the left frontal lobe of patient #3 (14 years old). Note that TH-ir neurons were located in the deep layers. **B** Higher magnification of the boxed area in **A**. Note the dense distribution of TH-ir fibers in layers I and II. **C** Higher magnification of the boxed area in **A**. **D** Example of staining of NPY-ir neurons (patient #3). **E** Example of staining of CCK-ir neurons in the left anterior temporal cortex of patient #4 (16 years old). **F**–**H** Group data showing the laminar distribution of TH-ir, NPY-ir, and CCK-ir neurons (black) and their density in each layer (blue). **I** Percentage of cells labeled by different markers in the total cortical neuron population (black, patients #1, 3, 4, 7, 8, and 9) and their density in gray matter (blue, patients #7–9). Error bars denote SEM.

Next, we investigated the distribution patterns of TH-ir, NPY-ir, and CCK-ir neurons (Fig. 3 and Tables 2, 3). TH-ir (Fig. 3A, B, C, F) and NPY-ir (Fig. 3D, G) neurons were distributed mainly in layers V and VI and the white matter, taking up 0.53% ± 0.14% (V and VI; *n* = 35,448) and 0.51% ± 0.06% (white matter; *n* = 26,859) of the total cortical neurons. The density of TH-ir and NPY-ir neurons in the gray matter were 3 ± 1 (*n* = 144) and 2 ± 1 per mm^2^ (*n* = 132), respectively. Most of the TH-ir neurons were bipolar or bitufted cells (Fig. 3A, C), and many NPY-ir neurons emitted two main dendrites in different directions (Fig. 3D). The TH and NPY antibodies also labeled a mass of long fibers in layer I and the white matter. In layer I, these fibers were horizontal and parallel to the pia. TH-ir fibers were present in the superficial layers (Fig. 2B). It should be noted that most of these TH fibers may come from subcortical dopaminergic or noradrenergic neurons [39].

CCK-ir neurons were concentrated in layers IV and VI, and a much lower density was observed in other layers. They constituted 4.38% ± 0.47% of all cortical neurons (*n* = 28,563), and their density in the gray matter was 23 ± 7 per mm^2^ (*n* = 1434). Because the neurites of CCK-ir neurons were only weakly labeled, their distribution pattern was unclear. The majority of CCK-ir neurons possessed a round or oval soma (Fig. 3E). Previous experiments have revealed that a subpopulation of pyramidal cells express CCK [7]. We also found a similar pattern (except for samples from the infant patient #1); however, the fluorescence immunosignals were relatively weaker than in non-pyramidal cells. Since we focused on cortical interneurons, we only included the strongly-labeled non-pyramidal neurons for data analysis.

### Co-localization of NPY, CCK, and TH with PV and SST

Although many molecular markers have been used to identity different interneuron types, none can actually define a cell type. Given that two or more molecular markers can be expressed in a single cell type, we next sought to clarify the overlap between PV-ir and SST-ir cells and those containing NPY-ir, CCK-ir, or TH-ir.

**Fig. 4:**
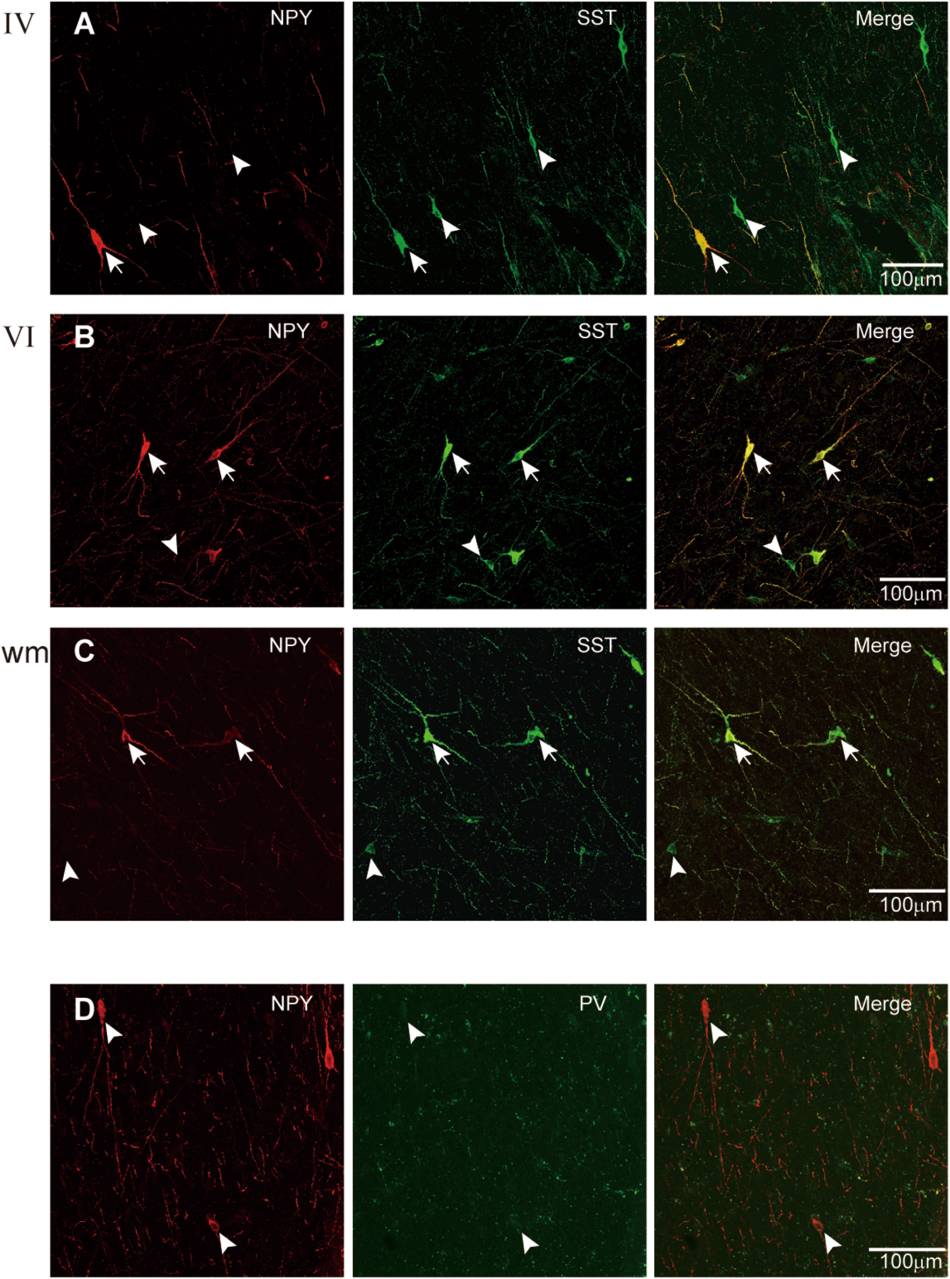
Co-expression of NPY with SST or PV. **A**–**C** Double-labeling for SST and NPY in layers IV (**A**), VI (**B**), and the white matter (**C**). Arrows indicate cells co-expressing NPY and SST; arrowheads indicate cells without co-expression. Note that NPY and SST were highly co-localized in layer VI and white matter cells. **D** Double-staining for NPY and PV showing that all the three NPY cells were PV-negative. Images were from tissues of patient #1 (1.3 years old).

Several distinct subpopulations of NPY-containing neurons have been reported in rat and monkey cortex, one of which co-expresses SST [49–51]. Consistent with these reports, our results revealed that 97.7% ± 1.5% of NPY-ir interneurons (*n* = 43) in epileptic human cortex also expressed SST, and these co-expressing neurons were 38.2% ± 3.1% of all SST-ir neurons (*n* = 110). Since NPY neurons were only present in layers IV, V, VI, and the white matter, the co-expressing neurons were mainly located in the deep layers and absent from the superficial layers (Fig. 4A–C). In sharp contrast, we found no co-localization between PV-ir and NPY-ir (Fig. 4D). In this particular experiment, we examined the co-expression in tissues from patients #1–6.

**Fig. 5:**
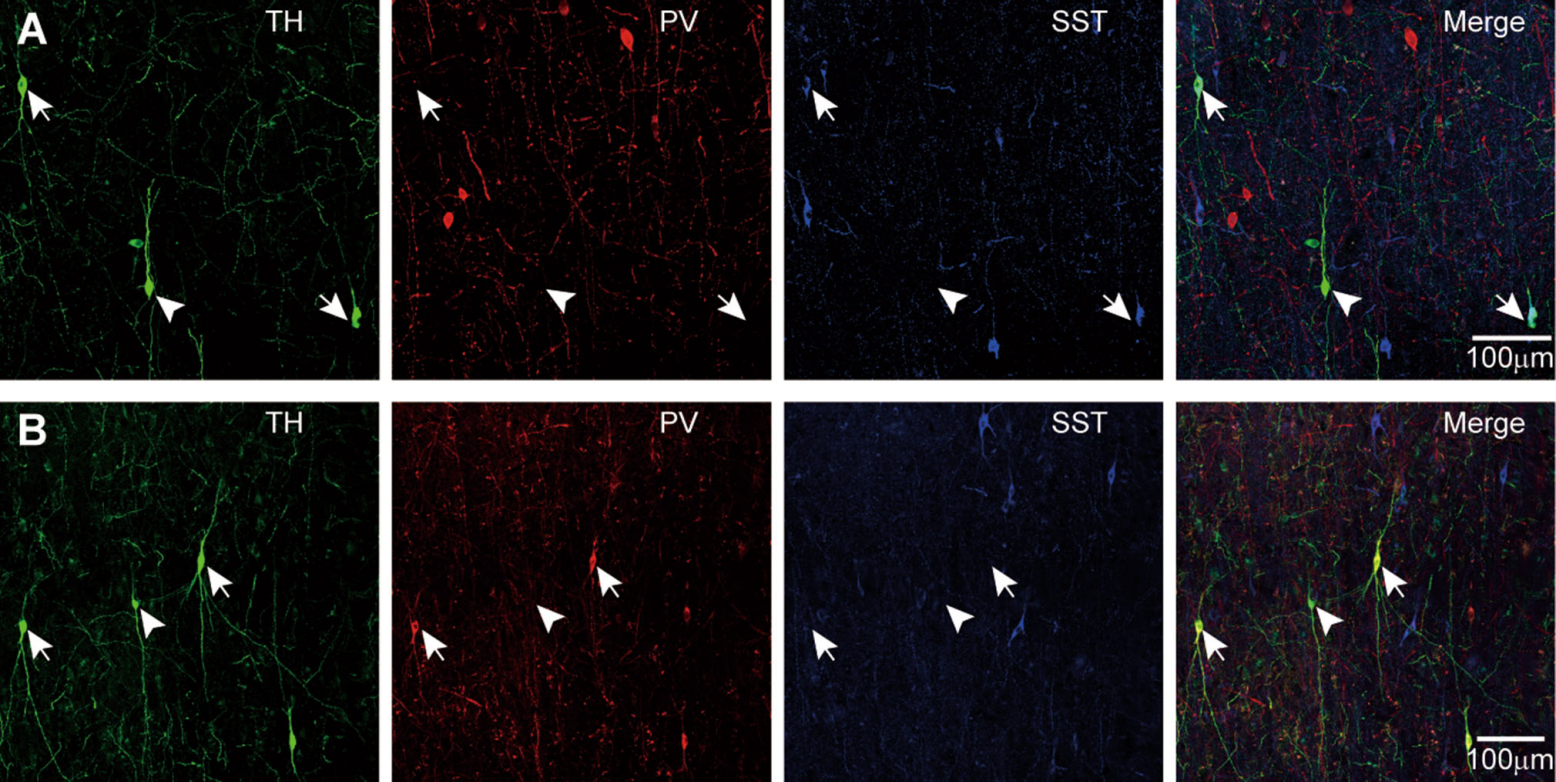
Co-expression of TH with PV and SST. **A** Triple-labeling for TH, PV, and SST in tissues from the left frontal lobe (patient #3, 14 years old). TH-positive cells were more likely to co-express SST. Arrows indicate cells with TH and SST co-expression; arrowheads point to a TH cell without SST or PV expression. **B** Labeling as in **A** but from patient #1 (right temporal lobe, 1.3 years old). TH-positive cells were more likely to co-express PV. Arrows point to a TH-ir neuron co-expressing PV, while arrowheads indicate a TH-ir neuron with no PV labeling.

Our experiments showed that 34.2% ± 7.3% of TH-ir neurons (*n* = 105) were SST- positive and 14.6% ± 5.6% TH-ir neurons (*n* = 82) were PV-positive (Table 6). In this part of work, we used the tissues from patients #1, 3, 4, and 5 (1, 3, and 4 for triple-staining, and 5 for double-staining). As shown in Table 5, the percentages of TH-positive cells co-expressing PV or SST varied between patients, possibly resulting from age differences. The staining shown in Fig. 4 was performed in tissues from patients #3 (Fig. 5A) and #1 (Fig. 5B).

**Table 6:** Percentages (mean ± SEM) of cells showing co-expression in the total population of PV, SST, TH, NPY, or CCK-positive cells

Neurons containing	Percentage showing co-localization				
	PV	SST	TH	NPY	CCK
PV	–	–	1.11% ± 0.33%	0	15.6% ± 3.1%
SST	–	–	9.30% ± 1.18%	38.2% ± 3.1%	0
TH	14.6% ± 5.6%	34.2% ± 7.3%	–	0	1.79% ± 0.67%
NPY	0	97.7% ± 1.5%	0	–	0
CCK	28.0% ± 5.4%	0	0.25% ± 0.09%	0	–

**Fig. 6:**
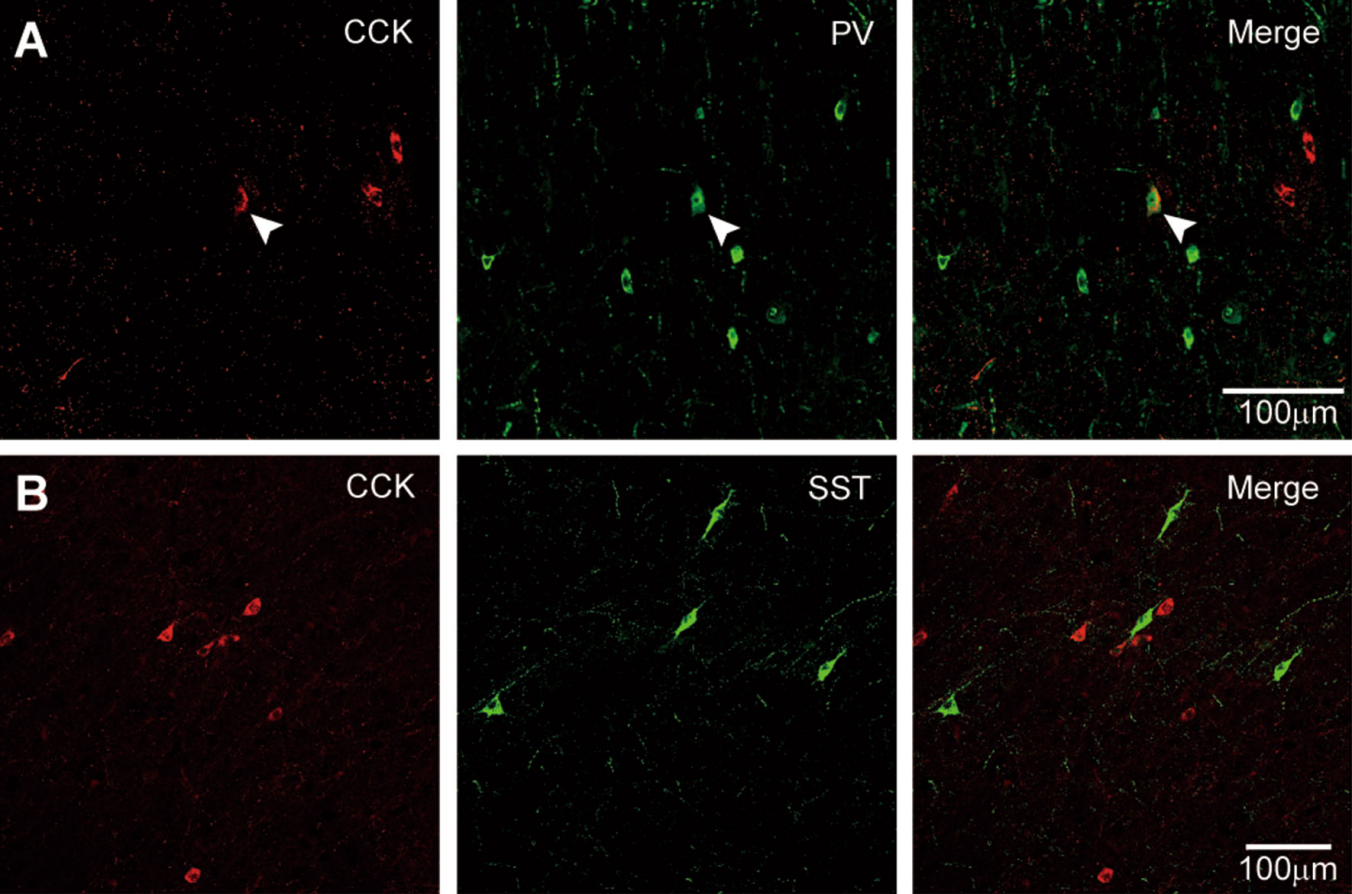
Co-expression of CCK with PV or SST. **A** Double-labeling for CCK and PV in the left anterior temporal cortex of patient #4 (16 years old). Arrowheads indicate a cell positive for both CCK and PV. Note the absence of PV-ir in the other two CCK-positive cells. **B** Double-labeling for SST and CCK in the left anterior temporal cortex of patient #2 (1.6 years old). Note the absence of co-expressing cells.

We found that 28.0% ± 5.4% of CCK-ir neurons (*n* = 321) co-expressed PV in double-staining experiments (Fig. 6A). Interestingly, although CCK-positive cells were distributed from layer II to the white matter, the CCK-PV-co-expressing cells were only found in layer IV. In addition, CCK-ir neurons were not labeled by the antibody to SST (Fig. 6B). We performed this double-staining in tissues from patients #2–4.

**Fig. 7:**
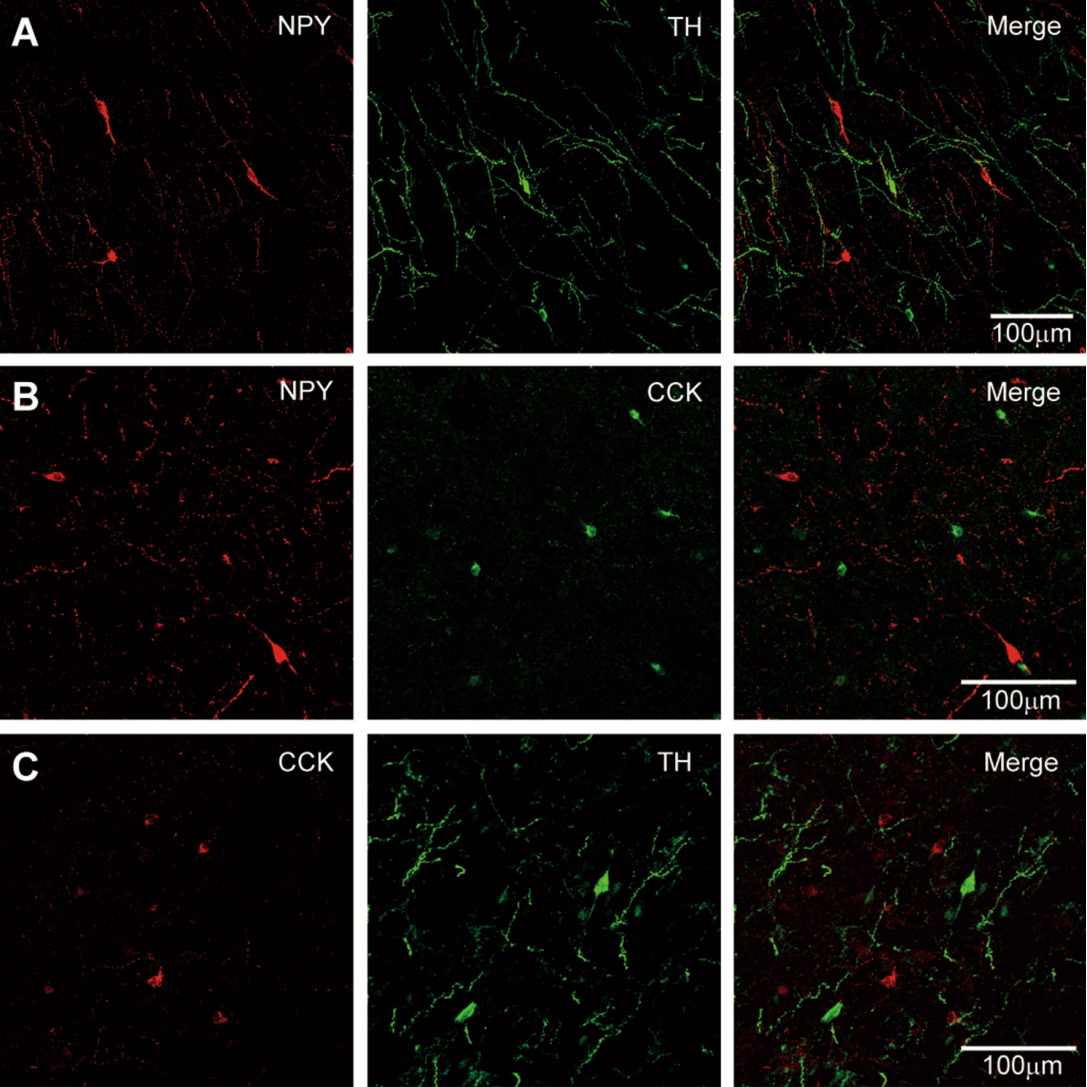
No overlap of TH-, NPY-, and CCK-positive cells. **A** Double-labeling for NPY and TH in the left anterior temporal lobe of patient #2 (1.6 years old). **B** Double-labeling for NPY and CCK in the left anterior temporal lobe of patient #6. **C** As in **B**, but with double-staining for TH and CCK (patient #6, 28 years old). Note the absence of cells co-expressing any two of the three markers.

We also examined the co-expression among NPY, TH, and CCK (Fig. 7) in double-labeling experiments. Although both TH-ir and NPY-ir neurons were located in deep layers and the white matter, we found no overlap between these cells (Fig. 7A). No co-localization between NPY and CCK (Fig. 7B) or TH and CCK (Fig. 7C) was found. These results suggest that these chemical markers identify distinct non-overlapping cell types in the neocortex. Tissues from patients #2, 4, and 6 were used in these experiments.

## Discussion

Although it is very important to understand the neural substrates of human cognitive functions, few studies have examined the cell types and their distribution in cortical circuits. Previous work in adult human cortical tissues have revealed the density and distribution patterns of certain types of neurons [25, 39, 52]. In this study, we investigated the cell density and laminar distribution of neurons expressing PV, SST, CCK, NPY, and TH in epileptic human cortex. Our results indicated that PV, SST, and CCK cells were located in cortical layers II to VI, while TH-ir and NPY-ir cells were preferentially located in deep layers from IV to the white matter. In addition, we found subpopulations of neurons labeled by two markers: PV+TH, PV+CCK, SST+NPY, and SST+TH. In contrast, we found no overlap between PV and NPY, SST and CCK, NPY and TH, NPY and CCK, and TH and CCK (Tables 5 and 6).

We used epileptic tissue to investigate the cell density and distribution of interneurons types. It remains to be further determined whether specific cell types are vulnerable to epileptic conditions. Early examination has revealed a loss of PV-positive cells in human cortical tissue [22] and the hippocampal CA1 region with temporal-lobe epilepsy [53]. However, it has also been reported that the percentage of PV-ir cells among all cortical neurons is relatively high (~ 14%), only slightly less than that of GABAergic neurons (~ 17%) in human epileptic cortex [54]. In non-epileptic human prefrontal cortex, PV-ir cells constitute ~ 20% of all GABAergic neurons [26]. If GABAergic neurons comprise 20%–30% of the total cortical neuron population [2, 3, 55, 56], the estimated percentage of PV-ir cells in the cortex would be 4%–6%. In agreement with this estimate, our results from non-epileptic human tissue revealed that ~ 7% of all cortical neurons showed PV-ir (data not shown), similar to that observed in epileptic tissue (7%, Fig. 2 and Table 4). In addition, the laminar distribution pattern of PV-ir neurons in epileptic tissue (Fig. 2 and Tables 2, 3) was also similar to that in non-epileptic tissue [26]. Since we chose to use tissue sections showing clear stratification (from layer I to the white matter) without any structural abnormality, they represented relatively normal tissue surrounding the epileptogenic zone. Indeed, the PV-ir cell density (~ 34 per mm^2^, Table 3) was comparable to that in non-epileptic peri-tumor tissue in our experiments, and similar to the PV density in cortical tissue without abnormal spiking (35 per mm^2^) [25] and cryptogenic tissue (~ 42 per mm^2^) [57]. However, the density was higher than that in the epileptogenic zone of epilepsy patients (~ 20 per mm^2^) [57], suggesting that only PV neurons in the epileptogenic zone are vulnerable to epileptic conditions.

Distinct from PV-ir cells, however, other cell types might be vulnerable to epileptic conditions. The cell densities of SST-ir and NPY-ir neurons in relatively normal tissue have been reported as ~ 40 and 17 per mm^2^, respectively [25], much higher than in our experiments (16 and 2 per mm^2^, Table 3), suggesting a substantial reduction of SST-ir and NPY-ir cells in epileptic conditions. Previous studies have shown that NPY neurons are widely distributed throughout the cortex but more frequently in layers II, III, and IV [51, 58, 59]. However, our results revealed that NPY neurons were mainly present in deep layers (IV, V, and VI) and the white matter. A similar deep-layer distribution pattern of TH-ir cells was observed in our immunostaining experiments, consistent with previous findings in human tissue [37, 39, 60]. CCK-ir cells may also be subject to alterations in epileptic conditions. The CCK mRNA expression level in the rodent cortex increases after multiple consecutive kindled seizures [61]. In our experiments, we used tissues from infant, teenage, and adult patients (#1, 3, 4, 7, 8, and 9; Table 1) and found, in general, no clear difference in cell proportions and laminar distribution patterns for most of the markers, suggesting that cell distribution does not change during early development. The proportion of TH-positive cells in the whole population of cortical neurons in the infant patient was relatively lower, possibly due to the high neuronal density in early developmental stages. Moreover, we found that the percentages of positive cells in all cortical neurons showed little difference between patients with different durations of epilepsy (Table 1).

In the co-expression experiments, we found that NPY-ir cells did not express PV, TH, or CCK, and CCK cells did not express SST and TH. Previous studies in rodents have shown that only a subpopulation of NPY neurons express SST [49–51]; our results indicated, however, that most NPY cells were positive for SST but negative for PV. We also found that subpopulations of TH cells also expressed PV or SST. In these experiments, we used tissues from different brain regions including the frontal or temporal lobes. Apart from the infant patient (#1) who showed distinct co-expression patterns between TH and PV or SST, we found no evident difference in the co-expression of different markers, indicating a similar co-expression pattern across different brain regions.

Together, our results revealed the distribution pattern and the overlap of biochemically-identified cortical GABAergic neurons. The results also suggested that the density and laminar distribution of PV-ir cells are largely preserved in epileptic tissue. However, whether other interneuron types (e.g. SST, CCK, NPY, and TH) change in response to epileptic seizures remains to be further examined. In addition to the markers used in the current study, non-pyramidal GABAergic neurons in the cortex also express other neuropeptides, calcium-binding proteins, and neurotransmitter receptor markers, such as vasoactive intestinal polypeptide, nitric oxide synthase, calretinin, calbindin, and 5-hydroxytryptamine 3a receptor. The distribution patterns of these neurons in the human cortex need to be examined in future studies.

## Electronic supplementary material

Below is the link to the electronic supplementary material.

Supplementary material 1 (PDF 81 kb)
