# Supplementary material for: Laminar Distribution of Neurochemically-Identified Interneurons and Cellular Co-expression of Molecular Markers in Epileptic Human Cortex
Source: Neurosci Bull. 2018 Aug 31;34(6):992–1006. doi: 10.1007/s12264-018-0275-x (PMC6246828; doi:10.1007/s12264-018-0275-x)
Supplement: Supplementary file 1 — Supplementary material 1 (PDF 81 kb) [file 12264_2018_275_MOESM1_ESM.pdf]

Electronic Supplementary Material

Laminar Distribution of Neurochemically-Identified Interneurons and Cellular Co-expression of Molecular Markers in Epileptic Human Cortex

Qiyu Zhu[2], Wei Ke[1], Quansheng He[1], Xiongfei Wang[3], Rui Zheng[1], Tianfu Li[3], Guoming Luan[3], Yue-Sheng Long[4], Wei-Ping Liao[4], Yousheng Shu[1]

[1] State Key Laboratory of Cognitive Neuroscience and Learning & IDG/McGovern Institute for Brain Research, Beijing Normal University, Beijing 100875, China

[2] College of Pharmaceutical Sciences, Brain Institute, Capital Medical University, Beijing 100069, China

[3] Department of Neurosurgery, Epilepsy Center, Sanbo Brain Hospital of Capital Medical University, Beijing Key Laboratory of Epilepsy, Epilepsy Institution, Beijing Institute for Brain Disorders, Beijing 100093, China

[4] Institute of Neuroscience and Department of Neurology of the Second Affiliated Hospital of Guangzhou Medical University; Key Laboratory of Neurogenetics and Channelopathies of Guangdong Province and the Ministry of Education of China, Guangzhou 501260, China

Table S1. Primary and secondary antibodies

Antibodies	Dilution	Source	Cat. #
Anti-NeuN (Mouse)	1:500	Millipore	MAB377
Anti-Parvalbumin (PV) (Goat)	1:500	Swant	PVG214
Anti-Parvalbumin (PV) (Mouse)	1:500	Millipore	MAB1572
Anti-Somatostatin (SST) (Goat)	1:200	Santa Cruz	SC7819
Anti-Neuropeptide Y (NPY) (Rabbit)	1:500	ImmunoStar	22940
Anti-Tyrosine Hydroxylase (TH) (Rabbit)	1:500	Millipore	AB152
Anti-Tyrosine Hydroxylase (TH) (Mouse)	1:500	Millipore	MAB318
Anti-Cholecystokinin (CCK) (Mouse)	1:500	CURE Center,	AB9303

		UCLA	
Alexa 488 anti-rabbit (Donkey)	1:1000	Invitrogen	A21206
Alexa 555 anti-mouse (Donkey)	1:1000	Invitrogen	A31570
Alexa 647 anti-goat (Donkey)	1:1000	Invitrogen	A21447
